# Salt and salted food intake and subsequent risk of gastric cancer among middle-aged Japanese men and women

**DOI:** 10.1038/sj.bjc.6601511

**Published:** 2004-01-06

**Authors:** S Tsugane, S Sasazuki, M Kobayashi, S Sasaki

**Affiliations:** 1 Epidemiology and Biostatistics Division, National Cancer Center Research Institute East, 6-5-1 Kashiwanoha, Kashiwa, 277-8577, Japan

**Keywords:** diet, prospective studies, salts, stomach neoplasms

## Abstract

Evidence on the association between salt intake and gastric cancer is sparse, especially in prospective studies. We conducted a population-based prospective study in Japan, where the majority of men has been infected with *Helicobacter pylori*. A total of 18 684 men and 20 381 women aged 40–59 years who reported their dietary habits and did not report any serious disease at baseline were followed from 1990 to 2001. A total of 486 cases, 358 men and 128 women, with histologically confirmed gastric cancer were documented among them. The quintile category of salt intake was dose-dependently associated with gastric cancer risk in men after adjusting for potential confounding factors (*P* for trend <0.001), while a trend was not clear in women (*P* for trend=0.48). Although stratification by study area, with varied salt intake and gastric cancer incidence, attenuated the observed clear associations with salt and salted foods, the frequency categories of highly salted foods such as salted fish roe and salted fish preserves were strongly associated with the risk in both sexes. Restriction of salt and salted food intake is a practical strategy to prevent gastric cancer in areas with high risk.

Gastric cancer is the second most frequent cause of cancer deaths worldwide, with an estimated 776 000 deaths in 1996, almost two-thirds of which were in developing countries (World Health Organization, 1997). In Japan, gastric cancer killed 50 000 people in 2000, which was the second most frequent cancer death (17% of all cancer deaths; Ministry of Health, Labor and Welfare, Japan). Moreover, a total of 100 000 new cases of gastric cancer were estimated in Japan in 1996, and it was the most common cancer site (21% of all incident cancers; Research Group for Population-based Cancer Registration in Japan, 2002). Therefore, prevention of gastric cancer is one of the most important elements in any cancer control strategy both in Japan and around the world.

Although salt and salted foods are probable risk factors, based on evidence from a large number of case–control and ecological studies (Tsugane *et al*, 1992b; Joossens *et al*, 1996; Kono and Hirohata, 1996), evidence from prospective investigation is scarce and inconsistent (Nomura *et al*, 1990; Kneller *et al* 1991; Kato *et al*, 1992). The recent report of a joint WHO/FAO Expert Consultation concluded that ‘Salt-preserved foods and salt probably increase the risk of stomach cancer’ (World Health Organization, 2003). The difficulties in estimating the intake of salt *per se* may account for divergent findings.

We tested this hypothesis in Japan in a population-based prospective study in four public health centre areas with varying gastric cancer mortality rates and where the majority (75%) of randomly selected men aged 40–49 years has been infected with *Helicobacter pylori* (Tsugane *et al*, 1994).

## MATERIALS AND METHODS

### Study cohort

The Japan Public Health Center-based prospective study on cancer and cardiovascular diseases (JPHC Study) Cohort I, which was partly reported elsewhere (Tsugane *et al*, 1999), provided a basis for this investigation. As of 1 January 1990, we established a population-based cohort of 54 498 people (27 062 men and 27 436 women) who resided in 14 administrative districts supervised by four regional public health centres (PHCs): 12 291 from Ninohe City and Karumai Town in the Ninohe PHC area of Iwate prefecture, 15 782 from Yokote City and Omonogawa Town in the Yokote PHC area of Akita, 12 219 from eight districts of Minami-Saku county in the Saku PHC area of Nagano and 14 206 from Gushikawa City and Onna Village in the Ishikawa PHC area of Okinawa. All were born between 1930 and 1949 (40–59 years of age at the baseline). These PHC areas were selected to represent the extent of variation in the mortality rate of stomach cancer based upon our previous ecological study (Tsugane *et al*, 1992a; Tsubono *et al*, 1997). The institutional review board of the National Cancer Center approved this study.

### Baseline survey

In 1990, a self-administered questionnaire was distributed to all registered residents. They were asked to report on their sociodemographics, personal medical history, smoking and drinking history and diet. A total of 43 149 subjects, 20 665 men (76%) and 22 484 women (82%), returned their questionnaires. Although the date of questionnaire completion ranged from January 1990 to May 1992, 54% responded between February 1990 and March 1990. Only 4% were completed after October 1990.

The weekly intake frequency of 27 food items was reported in four categories: rarely, 1–2 days week^−1^, 3–4 days week^−1^ and almost daily (5+ days week^−1^). For rice, miso soup and nine kinds of beverage, the daily amount consumed was also reported. Design and participation rate of the baseline survey and dietary habits among JPHC study participants at baseline survey were reported elsewhere (Tsugane and Sobue, 2001; Tsugane *et al*, 2001).

Based on dietary record data, we developed a food composition table that corresponded to the food items listed in the questionnaire and determined standard portions/units for each food category using the observed median values. The food composition table was developed and standard portions/units were assigned separately for men and women. Daily nutrient intakes were calculated by multiplying the consumption frequency of each food by the nutrient content of the standard portions/units and summing these values for all foods. Calculation of total energy and 30 nutrients was done with the Standard Tables of Food Composition (Science and Technology Agency, 1982) and other sources. The validity was assessed among subsamples (94 men and 107 women) with actual nutrient intake, which was calculated based on a 28-day dietary record (7 consecutive days in four seasons; 14 days in the Ishikawa PHC area; Tsugane *et al*, 2003). The Spearman rank correlations between two indices for sodium intake were 0.49 in men and 0.54 in women (Tsubono *et al*, 2003). Those with two-time 24-h urine excretion level (32 men and 57 women, except Ishikawa PHC area) were 0.38 in men and 0.12 in women (Tsubono *et al*, 2003).

The following food items were considered as salted food in our analysis: miso soup (salt content: 0.5–1.2%), pickled vegetables (1–10%; specific foods include shiozuke, with 1.7% in Chinese cabbage, 2.8% in cucumber, and nuka-misozuke, with 1.7% in eggplant, 2.8% in cucumber), salted fish roe (tarako, salted Alaska pollack roe, 6.6%, suziko or ikura, salted salmon roe, 9.7%), salted fish preserves (shiokara, salted fish guts, 11.4% in squid, neri-uni, salted gonad paste of sea urchin, 11.9%) and dried or salted fish (mezashi, salted and semidried skewered sardine, 4%, and shio-sake, salted salmon, 8%). Salt content data for specific food items were taken from with Standard Tables of Food Composition (Science and Technology Agency, 1982). The Spearman rank correlations with the dietary records (g day^−1^) were 0.41 for miso soup, 0.67 for pickled vegetables, 0.61 for salted fish roe, 0.43 for salted fish preserves and 0.55 for dried or salted food in men, and 0.49, 0.61, 0.47, 0.34 and 0.33 in women, respectively (Tsugane *et al*, 2003). Miso soup was categorised into the following four groups in this analysis: not daily, 1 cup day^−1^, 2 cups day^−1^, 3+ cups day^−1^.

We excluded subjects with a self-reported serious illness (cancer, cerebrovascular disease, myocardial infarction, or chronic liver disease) at baseline as well as non-Japanese subjects and subjects who had already moved away at the baseline, which we confirmed during the follow-up period. We further excluded subjects who reported extreme total energy intake (upper 2.5% or lower 2.5%). These exclusions left 18 684 eligible men and 20 381 women included in this study.

### Follow-up and identification of gastric cancer

#### Death and change in residence

We followed all registered cohort subjects from 1 January 1990 to 31 December 2001. In Japan, all death certificates are submitted to a local government office and forwarded to the PHC in the area of residence. Mortality data are then sent to the Ministry of Health and Welfare and coded for inclusion in the National Vital Statistics. The registration of deaths in Japan is required by the Family Registration Law and is believed to be complete. Therefore, all deaths of cohort subjects were based upon death certificates from each PHC, whenever they stayed in their original area. The changes in residence status were identified annually through the residential registry in each area.

#### Cancer registry for JPHC study

Newly diagnosed cases of cancer were reported by hospitals in and around the study areas when the birth date and residence fulfilled cohort inclusion criteria. Candidate patients were linked by name, address, and date of birth and entered in the cancer registry for the JPHC Study Cohort I. In the Ninohe and Ishikawa PHC areas, a prefecture-wide cancer registry was available (i.e. Iwate and Okinawa Prefecture cancer registry, respectively). Death certificates were used as a supplementary information source for the cancer registry, and 259 cases were first notified by it. As of July 2002, such cases accounted for 7.6% (DCN (Death Certificate Notification) %) of all the 3429 entries, which had been diagnosed in 1990–2001. Among them, 39 cases were not confirmed by medical records, and they would account for 1.1% (DCO (Death Certificate Only) %) of all the entries.

#### Identification of gastric cancer

Cases of gastric cancer were extracted from the cancer registry for the JPHC Study, based on site (International Classification of Diseases for Oncology (ICD-O) code: C160–169) and histological confirmation by biopsy or surgery. A total of 486 cases of gastric cancer, 358 in 18 684 men and 128 in 20 381 women, were documented as of July 2002 with a histologically proven diagnosis in 1990–2001. This left 10 cases without histological confirmation, which we considered not diagnosed with gastric cancer.

Cardia cancer was defined as a tumour located in the oesophagogastric junction or upper third of the stomach (International Classification of Diseases for Oncology (ICD-O) code C160–161; World Health Organization 1990). A tumour located in the lower side of the stomach was classified as distal gastric cancer (ICD-O code C162–167). Those subsites that could not be classified for its diffuse lesion (ICD-O code C168) or those with no information (ICD-O code C169) were categorised as unclassified. Histological subdivisions were made according to Lauren’s (1965) classification.

### Statistical analysis

Person-years at risk were calculated from 1 January 1990 to the date of diagnosed gastric cancer, the date of death, or change in residence or 31 December 2001, whichever came first. A Cox's proportional-hazards model was used to calculate the relative risk (RR) by category of each frequency of dietary intake at baseline after adjusting for covariates using PROC PHREG of the SAS program (SAS Institute, Inc., Cary, NC, USA). Cigarette smoking, fruit and non-green–yellow vegetable intake and age were principally adjusted based on our previous findings (Kobayashi *et al*, 2002; Sasazuki *et al*, 2002). Considering the observed colinearity of the intake frequency of three kinds of vegetables (e.g. green, yellow and other vegetables), only non-green–yellow vegetable intake was included in the multivariate model, because it was more clearly associated with gastric cancer risk (Kobayashi *et al*, 2002). Although analyses by histological type and by anatomical subsite were conducted, the results are not shown because of unstable findings due to reduced power.

## RESULTS

Age-adjusted incidence rates per 100 000 person-years with number of subjects, person-years of follow-up and number of gastric cancer cases are shown by sex and study area in Table 1

**Table 1:** Number of subjects and incident gastric cancer by sex and study area

**Sex and study area**	**Number of subjects**	**Person-years of follow-up**	**Number of incident gastric cancers**	**Age-adjusted incidence rate per 100 000 person-years**	**Anatomic subsite cardia/distal/unknown**	**Histological type differentiated/undifferentiated/unclassified**
*Men*	18 684	207 110	358	173	42/250/66	218/117/23
Ninohe, Iwate	3930	43 080	43	104	4/23/16	25/17/1
Yokote, Akita	5046	56 665	168	295	22/126/20	100/63/5
Saku, Nagano	5034	56 016	118	213	15/82/21	84/26/8
Ishikawa, Okinawa	4674	51 349	29	55	1/19/9	9/11/9
						
*Women*	20 381	232 463	128	55	12/96/20	47/71/10
Ninohe, Iwate	4602	52 484	18	35	2/10/6	9/9/0
Yokote, Akita	5858	67 506	57	84	5/46/6	23/31/3
Saku, Nagano	5135	58 912	36	61	4/26/6	11/22/03
Ishikawa, Okinawa	4786	54 061	17	31	1/14/2	4/9/4

, as well as the proportion of each anatomic subsite and histological type. Those rates were three times higher in the men of all three PHC areas except Ishikawa, with similar rates, and differed substantially by PHC area, especially among men. There were six- and two-fold differences with regard to study area in men and women, respectively. Most tumours (70% in men, 75% in women) were located in the distal portion, and there were no significant differences according to sex or study area. As to histological type, the differentiated type of gastric cancer was predominant in men (61%), whereas it was less common in women (37%). Among men, the proportion of differentiated type was slightly higher in the Saku PHC area and lower in Ishikawa PHC area.

The baseline characteristics according to each quintile category of salt intake are shown in Table 2

**Table 2:** Background characteristics of subjects by quintiles of salt intake

	**Men (*n*=18 684)**					**Women (*n*=20 381)**				
**Quintile**	**1 (low)**	**2**	**3**	**4**	**5 (high)**	**1 (low)**	**2**	**3**	**4**	**5 (high)**
**Median (g/d)^a^**	**2.9**	**4.8**	**6.1**	**7.5**	**9.9**	**2.6**	**4.2**	**5.3**	**6.4**	**8.2**
*Study area* (%)										
Ninohe, Iwate	8	15	21	27	35	10	16	24	30	33
Yokote, Akita	13	23	29	33	37	15	24	31	34	39
Saku, Nagano	19	28	32	32	22	19	30	29	27	21
Ishikawa, Okinawa	60	33	17	8	6	56	29	16	9	6

Age (years)	48.7	49	49.4	49.9	50.1	48.9	49.3	49.7	50	50

Current smokers (%)	49	51	54	56	56	8.2	6.5	4.7	3.6	4.2

Daily alcohol drinkers (%)	25	34	42	46	47	2.1	2.7	1.8	2	2.2

*Salted food intake*										
Miso soup (bowls week^−1^)	4.1	8.3	12.2	15.2	20.6	3.5	6.8	10.8	13.8	17.3
Pickled vegetables (days week^−1^)	1.4	2.8	3.9	4.7	5.1	1.8	3.4	4.3	5	5.4
Salted fish roe (days week^−1^)	0.4	0.8	1.1	1.5	2.2	0.4	0.9	1.1	1.4	2.1
Salted fish preserves (days week^−1^)	0.2	0.5	0.8	1.1	1.7	0.2	0.4	0.5	0.7	1.2
Dried or salted fish (days week^−1^)	0.9	1.5	1.9	2.3	2.9	1.1	1.8	2.1	2.4	2.9

*Fruit and vegetables*										
Fruit (days week^−1^)	2.3	2.9	3.1	3.4	3.5	3.2	4	4.3	4.5	4.5
Green vegetables (days week^−1^)	3.2	3.4	3.6	3.6	4	3.6	3.9	4	4.1	4.2
Yellow vegetables (days week^−1^)	2.4	2.6	2.7	2.8	2.9	3.1	3.3	3.4	3.5	3.5
Other vegetables (days week^−1^)	3	3.5	3.9	4.1	4.4	3.5	4.1	4.4	4.6	4.7

a Based on food frequency questionnaire.

. Approximately 60% of Ishikawa subjects were categorised in the lowest quintile, whereas the majority of Ninohe and Yokote subjects were located in the upper two quintiles. Smokers were more common in upper quintiles of salt intake in men, but they were more common in lower quintiles in women. The intake frequencies of five salted foods were closely correlated with salt intake. The frequencies of fruit and three kinds of vegetable intake were also positively correlated with salt intake, although not remarkable.

The Relative risks and 95% confidence interval (CI) of each quintile category of salt intakes are shown in Table 3

**Table 3:** Multivariate relative risks (RR) of incident gastric cancer by quintiles of salt intake at baseline in men and women: JPHC Study, 1990–2001

	**Quintiles of salt intake**					
	**1 (low)**	**2**	**3**	**4**	**5 (high)**	***P* for trend**
*Men*						
Person-years of follow-up	40 655	41 445	41 165	41 831	42 016	
No. of incident cancers	35	63	73	95	92	
Age-adjusted incidence rate	93	158	179	216	209	
Multivariate RR^a^	1	1.74	1.96	2.3	2.23	
95% CI	Reference	1.14–2.66	1.30–2.97	1.53–3.46	1.48–3.35	<0.001
Multivariate RR^b^	1.00	1.36	1.35	1.54	1.5	
95% CI	Reference	0.89–2.09	0.88–2.07	1.01–2.34	0.98–2.29	0.08

*Women*						
Person-years of follow-up	45 828	46 196	46 811	47 097	47 032	
No. of incident cancers	23	22	26	19	38	
Age-adjusted incidence rate	52	48	55	39	77	
Multivariate RR^a^	1	0.86	0.96	0.58	1.32	
95% CI	Reference	0.47–1.56	0.54–1.72	0.30–1.12	0.76–2.28	0.48
Multivariate RR^b^	1.00	0.75	0.81	0.48	1.09	
95% CI	Reference	0.41–1.37	0.45–1.48	0.25–0.95	0.61–1.94	0.85

a Adjusted for age in 1990 (40–44, 45–49, 50–54, 55–59 years), cigarette smoking (never, past, current) and fruit and non-green–yellow vegetable intake (almost none, 1–2 days week^−1^, 3–4 days week^−1^, almost everyday).

b Further stratified by PHC area.

CI=confidence interval.

; these were calculated after adjusting for age, cigarette smoking and fruit and vegetable intake. The quintile category of salt intake was dose-dependently associated with gastric cancer risk in men (*P* for trend <0.001) and it was attenuated after stratifying by study area (*P* for trend=0.08). Salt intake was not associated with gastric cancer risk in women.

RR and 95% CI of intake of five salted food categories are shown in Table 4

**Table 4:** Relative risks (RR) of incident gastric cancer by category of salted food intake at baseline in men: JPHC Study, 1990–2001

	**Category of intake**				
	**Not daily**	**1 cup day^−1^**	**2 cups day^−1^**	**3 or more cups day^−1^**	***P* for trend**
*Miso soup*					
Multivariate RR^a^	1	1.76	1.99	1.75	
95% CI	Reference	1.16–2.67	1.39–2.87	1.22–2.51	0.002
Multivariate RR^b^	1	1.51	1.37	1.01	
95% CI	Reference	0.98–2.32	0.90–2.10	0.64–1.61	0.29
Multivariate RR^c^	1	1.52	1.62	1.4	
95% CI	Reference	1.00–2.31	1.12–2.34	0.97–2.03	0.05

	**Almost none**	**1–2 days week^−1^**	**3–4 days week^−1^**	**Almost everyday**	
*Pickled vegetables (e.g. shiozuke, nuka-misozuke)*					
Multivariate RR^a^	1	1.54	2.71	2.35	
95% CI	Reference	0.97–2.46	1.76–4.19	1.57–3.54	<0.001
Multivariate RR^b^	1	1.4	2.28	1.86	
95% CI	Reference	0.87–2.24	1.45–3.59	1.19–2.89	0.008
Multivariate RR^c^	1	1.09	1.43	1.17	
95% CI	Reference	0.68–1.77	0.89–2.30	0.74–1.84	0.69

*Salted fish roe (e.g. tarako, suziko, ikura)*					
Multivariate RR^a^	1	1.58	2.18	2.44	
95% CI	Reference	1.23–2.04	1.60–2.97	1.41–4.23	<0.001
Multivariate RR^b^	1	1.42	1.94	2.21	
95% CI	Reference	1.09–1.85	1.39–2.70	1.24–3.92	<0.001
Multivariate RR^c^	1	1.02	1.29	1.45	
95% CI	Reference	0.78–1.34	0.92–1.80	0.83–2.56	0.08

*Salted fish preserves (e.g. shiokara, neri-uni)*					
Multivariate RR^a^	1	1.47	1.75	3.12	
95% CI	Reference	1.17–1.84	1.21–2.53	1.68–5.78	<0.001
Multivariate RR^b^	1	1.3	1.53	2.76	
95% CI	Reference	1.02–1.66	1.03–2.28	1.44–5.27	<0.001
Multivariate RR^c^	1	1.07	1.29	2.33	
95% CI	Reference	0.85–1.36	0.88–1.87	1.25–4.33	0.03

*Dried or salted fish (e.g. mezashi, shio-sake)*					
Multivariate RR^a^	1	2.16	1.85	2.23	
95% CI	Reference	1.53–3.05	1.26–2.70	1.37–3.63	0.007
Multivariate RR^b^	1	1.83	1.49	1.76	
95% CI	Reference	1.28–2.62	1.00–2.23	1.06–2.93	0.21
Multivariate RR^c^	1	1.15	0.94	1.2	
95% CI	Reference	0.79–1.70	0.62–1.43	0.71–2.02	0.74

a Adjusted for age in 1990 (40–44, 45–49, 50–54, 55–59 years), cigarette smoking (never, past, current) and fruit and non-green–yellow vegetable intake (almost none, 1–2 days ^−1^week, 3–4 days week^−1^, almost everyday).

b Further adjusted by quartile categories of salt intake.

c Further stratified by PHC area.

CI=confidence interval.

for men and Table 5

**Table 5:** Relative risks (RR) of incident gastric cancer by category of salted food intake at baseline in women: JPHC Study, 1990–2001

	**Category of intake**				
	**Not daily**	**1 cup day^−1^**	**2 cups day^−1^**	**3+ cups day^−1^**	***P* for trend**
*Miso soup*					
Multivariate RR^a^	1	1.15	0.82	1.11	
95% CI	Reference	0.66–2.01	0.49–1.38	0.67–1.84	0.65
Multivariate RR^b^	1	1.15	0.79	0.85	
95% CI	Reference	0.64–2.09	0.40–1.55	0.39–1.87	0.63
Multivariate RR^c^	1	1.11	0.76	1.05	
95% CI	Reference	0.64–1.94	0.45–1.29	0.62–1.79	0.52

	**Almost none**	**1–2 days week^−1^**	**3–4 days week^−1^**	**Almost everyday**	
*Pickled vegetables (e.g. shiozuke, nuka-misozuke)*					
Multivariate RR^a^	1	1.01	2.2	1.74	
95% CI	Reference	0.44–2.31	1.05–4.58	0.89–3.41	0.05
Multivariate RR^b^	1	1.08	2.52	2.01	
95% CI	Reference	0.47–2.47	1.18–5.37	0.97–4.17	0.03
Multivariate RR^c^	1	0.92	1.77	1.32	
95% CI	Reference	0.39–2.13	0.78–3.99	0.60–2.91	0.46

*Salted fish roe (e.g. tarako, suziko, ikura)*					
Multivariate RR^a^	1	1.49	1.58	3.5	
95% CI	Reference	0.98–2.28	0.91–2.76	1.62–7.56	0.003
Multivariate RR^b^	1	1.59	1.63	3.37	
95% CI	Reference	1.02–2.47	0.90–2.96	1.48–7.66	0.006
Multivariate RR^c^	1	1.26	1.26	2.67	
95% CI	Reference	0.79–2.00	0.68–2.32	1.18–6.07	0.07

*Salted fish preserves (e.g. shiokara, neri-uni)*					
Multivariate RR^a^	1	0.95	2.6	2.56	
95% CI	Reference	0.62–1.45	1.44–4.69	0.63–10.5	0.03
Multivariate RR^b^	1	0.94	2.43	2.29	
95% CI	Reference	0.60–1.46	1.29–4.55	0.55–9.59	0.06
Multivariate RR^c^	1	0.83	2.18	2.18	
95% CI	Reference	0.54–1.27	1.19–3.98	0.53–8.98	0.14

*Dried or salted fish (e.g. mezashi, shio-sake)*					
Multivariate RR^a^	1	1.43	1.66	1.22	
95% CI	Reference	0.81–2.52	0.92–3.03	0.53–2.82	0.31
Multivariate RR^b^	1	1.48	1.68	1.17	
95% CI	Reference	0.82–2.65	0.89–3.16	0.49–2.80	0.44
Multivariate RR^c^	1	1.05	1.17	0.89	
95% CI	Reference	0.54–2.05	0.58–2.37	0.35–2.22	0.98

a Adjusted for age in 1990 (40–44, 45–49, 50–54, 55–59 years), cigarette smoking (never, past, current) and fruit and non-green–yellow vegetable intake (almost none, 1–2 days week**^−1^**, 3–4 days week**^−1^**, almost everyday).

b Further adjusted by quartile categories of salt intake.

c Further stratified by PHC area.

CI=confidence interval.

for women; these were calculated after adjusting for age, cigarette smoking and fruit and vegetable intake. The frequency categories of all listed salted foods were closely associated with the risk in men (*P* for trend <0.05). Although the observed associations were attenuated after further adjusting for salt intake or further stratifying by study area, they were still evident for salted fish roe (*P* for trend <0.001 after adjusting for salt intake and 0.08 after stratifying by study area) and salted fish preserves (*P* for trend <0.001 and 0.03, respectively). For women, there were no clear associations for intake of miso soup, pickled vegetables and dried or salted fish. However, the associations for salted fish roe and salted fish preserves were clear (*P* for trend=0.003 for salted fish roe and 0.03 for salted fish preserves) and they were still evident even after further adjusting for salt intake (*P* for trend=0.006 for salted fish roe and 0.06 for salted fish preserves). These associations were still persistent, although not statistically significant, even after stratifying by study area.

## DISCUSSION

In this population-based prospective study of middle-aged Japanese men and women, we observed a dose-dependent increased risks of gastric cancer with the consumption of highly salted foods such as salted fish roe and salted fish preserves. In men, the associations were even clear for estimated total salt and the intake of other salted foods such as miso soup, pickled vegetables and dried or salted fish.

In experimental studies with rats, ingestion of salt is known to cause gastritis, and when coadministered, it enhances the carcinogenic effects of known gastric carcinogens such as *N*-methyl-*N*-nitro-*N*-nitrosoguanidine (MNNG; Tatematsu *et al*, 1975; Takahashi and Hasegawa, 1985). Moreover, many epidemiological studies showed that salt or salted food intake increased the risk of gastric cancer (Kono and Hirohata, 1996), including those conducted among Japanese (Tajima and Tominaga, 1985; Hoshiyama and Sasaba, 1992; Tsugane *et al*, 1992b). Intragastric high-salt concentration destroys the mucosal barrier, and leads to inflammation and damage such as diffuse erosion and degeneration. The induced proliferous change may enhance the effect of food-derived carcinogens (Takahashi and Hasegawa, 1985). Such mucosal damage also enhances *H. pylori* colonisation in mice (Fox *et al*, 1999) and possibly in humans (Tsugane *et al*, 1994), thereby inducing chronic gastritis (Blaser, 1990) and possibly increasing the risk of gastric cancer (International Agency for Research on Cancer, 1994).

The geographic difference observed in this prospective study confirmed our previous findings from an ecological study conducted in the identical study areas in 1989–1990 (Tsugane *et al*, 1991). The male age-standardised mortality rate (world population) per 100 000 population for 1985–1987 were 29.7 in Ninohe, 49.4 in Yokote, 42.8 in Saku and 17.2 in Ishikawa, respectively. In that study, average amount of salt excretion in 24-h urine (g day^−1^) from randomly selected men aged 40–49 years was closely associated (*r*^2^=0.995) with the gastric cancer mortality in these areas: 9.9 in Ninohe, 13.4 in Yokote, 11.9 in Saku and 8.0 in Ishikawa. However, this strong correlation was largely attenuated (*r*^2^=0.265) with average amounts of dietary salt intake (g day^−1^) based on 3-day dietary record and calculated from the Standard Tables of Food Composition (Science and Technology Agency, 1982): 15.5 in Ninohe, 15.0 in Yokote, 13.2 in Saku and 12.0 in Ishikawa. The average amount of estimated salt intake (g day^−1^) in this study with limited food items was based on the standard composition table (Science and Technology Agency, 1982): 7.7 in Ninohe, 7.3 in Yokote, 6.6 in Saku and 4.5 in Ishikawa. Therefore, the estimated salt intake was relatively overestimated in Ninohe and may have underestimated the true association between salt intake and gastric cancer incidence, especially in women. The precise estimation of salt intake may be implausible especially in different study areas, where the content of salt may be differed in each area even for the same food item. Although multiple 24-h urine collections may be an ideal method to estimate habitual salt intake (Liu *et al*, 1979), they are not feasible for a large-scale prospective study.

The observed weak link between salt intake and gastric cancer in women may also be due to relatively low validity of estimated salt intake (Tsubono *et al*, 2003). The Spearman rank correlation with 28-day dietary record was 0.54 in women, however, that with 2-day urinary excretion was only 0.12. The corresponding values in men were 0.49 and 0.38, respectively.

The associations with salt and salted food intake were always attenuated after stratifying by study area, where both salt/salted food intake and gastric cancer incidence varied significantly and were well correlated at the population level. Owing to the difficulties in estimating the intake of salt *per se*, its validity is expected to be lower within each study area with limited variation when compared with that in four areas combined. Therefore, stratification by study area may have underestimated the true association. Nonetheless, the associations were still clear with salt intake in men.

Among the salted foods, consumption of highly salted food such as salted fish roe and salted fish preserves were strongly associated with an increased risk of gastric cancer, even when adjusted for salt intake and stratified by study area both in men and women. The salt content of these salted foods is more than 5%; that of other salted foods such as miso soup, pickled vegetables and dried fish varied widely, but it is less than 5% in most foods. The observed findings might imply that either highly salted fish such as salted roe or salted preserves itself increased the risk of gastric cancer or that its intake was merely a good marker for a preference for salted foods or salt intake in general. Since ‘almost daily’ consumers of salted fish roe (2.2%) or salted fish preserves (0.9%) were limited, the intake frequency of these foods served to effectively categorise the subjects according to their preference for salted food intake. However, the intake frequencies of miso soup (77%) or pickled vegetables (49%) were relatively common, and their salt contents are different in each serving and each individual. Salt content of miso collected from 39 regions in 20 prefectures across Japan showed a wide variation, from 9.1 to 18.2% on the regional average (Watanabe *et al*, 1982) and those in miso soup preparation (18 g miso 200 g^−1^ soup in general recipe) varied individually. Salt contents of pickled vegetables also varied from less than 1% to over 10%. Even miso soup or pickled vegetables may have increased the risk of gastric cancer among subjects who generally consumed these types of foods with relatively higher salt content. An alternative explanation for the strong associations between highly salted foods and gastric cancer may be due to chemical caricinogens, which can be formed by reacting nitrate or nitrite in the process of preservation and of digestion in the stomach.

*Helicobacter pylori* infection is closely associated with the risk of gastric cancer (Huang *et al*, 1998; Helicobacter and Cancer Collaborative Group 2001). The prevalence of *H. pylori* IgG antibody among randomly selected men aged 40–49 years was 76% in Ninohe, 86% in Yokote, 72% in Saku and 63% in Ishikawa PHC areas in our previous ecological study in 1989–1990 (Tsugane *et al*, 1994), which nearly paralleled age-adjusted incidence rates of gastric cancer in each area (Table 1). However, consumption of salted fish roe and salted fish preserves in these male cohort subjects was also closely associated with gastric cancer incidence at the population level: 5.8 and 4.3 days month^−1^ in Ninohe, 7.9 and 4.9 days month^−1^ in Yokote, 4.3 and 3.8 days month^−1^ in Saku and 0.9 and 1.0 days month^−1^ in Ishikawa (Tsugane *et al*, 2001). Moreover, a cross-sectional analysis in our ecological study showed that the intake of both pickled vegetable and miso soup was associated with *H. pylori* infection (Tsugane *et al*, 1994), although salted fish roe and salted fish preserves were not assessed in that study. An infection with *H. pylori*, however, is itself unlikely to increase the intake of salted foods. Therefore, even if *H. pylori* infection causes gastric cancer, restriction of salt and salted food intake can at least reduce its risk. It should be acknowledged that the worldwide decrease in the age-adjusted incidence of gastric cancer was obviously not due to the intentional eradication of *H. pylori* infection, but can be related to a reduction in salted food intake by use of refrigerators (World Cancer Research Fund in association with American Institute for Cancer Research, 1997). However, it is possible that improvement of sanitary condition and familial crowding may have been associated with unintentional falls in the prevalence of *H. pylori* infection and consequently gastric cancer incidence in recent birth cohorts especially in developed countries (Taylor and Blaser, 1991; International Agency for Research on Cancer, 1994).

To summarise, the intake of salt and salted food, especially highly salted food, was closely associated with the risk of gastric cancer. Considering that a substantial number of Japanese consume such foods daily and the relatively high risk involved, the restriction of these foods may be a practical way to prevent gastric cancer.

## Appendix

The investigators and participating institutions in the JPHC Study Cohort I Group, a part of JPHC Study Group (Principal investigator: S Tsugane), were as follows: S Tsugane, S Sasaki, Epidemiology and Biostatistics Division, National Cancer Center Research Institute East, Kashiwa; J Ogata, S Baba, National Center for Circulatory Diseases, Suita; K Miyakawa, F Saito, A Koizumi, Iwate Prefectural Ninohe Public Health Center, Ninohe; Y Miyajima, N Suzuki, S Nagasawa; Akita Prefectural Yokote Public Health Center, Yokote; H Sanada, Y Hatayama, F Kobayashi, H Uchino, Y Shirai, T Kondo, Nagano Prefectural Saku Public Health Center, Saku; Y Kishimoto, E Takara, M Kinjo, T Fukuyama, Okinawa Prefectural Ishikawa Public Health Center, Ishikawa; S Matsushima, S Natsukawa, Saku General Hospital, Usuda; S Watanabe, M Akabane, Tokyo University of Agriculture, Tokyo; M Konishi, Ehime University, Matsuyama; S Tominaga, Aichi Cancer Center Research Institute, Nagoya; M Iida, S Sato, Center for Adult Diseases, Osaka; the late M Yamaguchi, Y Matsumura, National Institute of Health and Nutrition, Tokyo; Y Tsubono, Tohoku University, Sendai; H Iso, Tsukuba University, Tsukuba; H Sugimura, Hamamatsu University, Hamamatsu and M Kabuto, National Institute for Environmental Studies.
